# Cognitively Engaging Physical Activity for Targeting Motor, Cognitive, Social, and Emotional Skills in the Preschool Classroom: The Move for Thought preK-K Program

**DOI:** 10.3389/fpsyg.2021.729272

**Published:** 2021-11-29

**Authors:** Spyridoula Vazou, Myrto F. Mavilidi

**Affiliations:** ^1^Department of Kinesiology, Iowa State University, Ames, IA, United States; ^2^Early Start/School of Education, University of Wollongong, Keiraville, NSW, Australia

**Keywords:** integrated learning, early education, attention, behavioral control, perceived competence, hot executive functions, cool executive functions

## Abstract

Despite the growing body of research indicating that integrated physical activity with learning benefits children both physically and cognitively, preschool curricula with integrated physical activities are scarce. The “Move for Thought (M4T) preK-K” program provides activities on fundamental motor skills that are integrated with academic concepts, executive function, and social-emotional skills in the preschool environment. The aim of this study was to evaluate the feasibility, usability, and effectiveness of the M4T preK-K program over an eight-week period in 16 preschool centers (*N*=273; *M*_age_=4.22 *SD*=0.61) that were randomly assigned to the intervention (8 M4T; *n*=138; *M*_age_=4.31 *SD*=0.61) and the control (8 traditional; *n*=135; *M*_age_=4.13 *SD*=0.60) group. In both groups, teacher ratings of children’s attention, behavioral control, and social skills (i.e., cooperation, assertion, and self-control) in the classroom, as well as children’s perceived motor skill competence and executive functions, were collected before and after the intervention. A daily teacher log measured intervention fidelity and perceived experiences with the program. Results showed a significant improvement on attention scores for children in the M4T preK-K group, compared to the control group. No significant differences emerged for behavioral control, social skills, executive functions, and perceived motor competence among groups. A significant time effect was evident for executive functions, with both groups improving over time. Further, the program was well-received, easy to implement in the preschool classroom and with high rates of satisfaction for both children and teachers. The M4T preK-K program is promising in helping teachers prepare preschool children for future educational success.

## Introduction

Pioneering research has placed training of goal-directed behaviors responsible for thinking, acting, and problem-solving (i.e., executive function skills) as the main target areas for young children’s cognitive development (Diamond and Lee, 2011). Students who learn to acknowledge and regulate emotions, form positive relationships, work well with their peers, and deal effectively with conflict, exhibit stronger executive function skills and self-regulation, and thrive in the school environment (Denham and Brown, 2010; Eisenberg et al., 2010; Nadeem et al., 2010). A bidirectional developmental model argues that brain areas linked with executive functions reciprocally interact with those areas underlying attention control, stress physiology, and emotion (Blair and Ursache, 2011). Notably, executive functions and self-regulation are both influenced by experience and have been shown to predict academic performance in later school years (Bull and Scerif, 2001; Bull et al., 2008; Blair and Ursache, 2011; Durlak et al., 2011).

However, recent evidence distinguishes between cool (i.e., cognitive) and hot (i.e., affective) executive functions corresponding to different neural trajectories in the prefrontal cortex (Leshem et al., 2020). Hot executive functions are associated with the orbitofrontal cortex, anatomically suited for the integration of affective and non-affective information, and regulation of motivated responses (Happaney et al., 2004). Thus, they are addressing social and emotional skills, based on emotion regulation (Garon, 2016; Pesce et al., 2020). Cool executive functions are linked to the lateral prefrontal cortex and can be elicited by abstract, decontextualized problems, and affective neutral conditions (e.g., sorting by shape or size; Pesce et al., 2020). The core executive function skills consist of inhibition (i.e., the ability to stay focused and resist temptations), working memory (i.e., the ability to information in mind while mentally working with it), and cognitive flexibility (i.e., the ability to easily and quickly switch focus of attention; Diamond, 2013).

A recent meta-analysis found that training executive function skills can be more effective and enjoyable for children when is embedded in everyday activities, such as constantly challenging games (Takacs and Kassai, 2019). For example, the “Tools of Mind” program, initially, was based on Vygotski’s theory and in particular on the notion that learning promotes cognitive development when it occurs within a sociocultural context (Bodrova and Leong, 2006). This program has been expanded nowadays primarily in private education, known as Montessori pedagogy, offering activities for promoting both cool and hot executive functions, including self-regulatory private speech (e.g., telling yourself what to do), dramatic play and aids to facilitate memory and attention. It was found that preschool children in the intervention group that used the Tools of the Mind program had highest scores in executive function tasks, especially the most demanding ones, than children in the control group (Diamond et al., 2007). Practices targeting social and emotional learning, such as children following directions, taking turns and sharing, persisting at challenging tasks, creating greater enjoyment for school, and paying attention, have been identified in the literature as vital for school programs to be effective, especially if those qualities are also integrated with academic learning (Bierman et al., 2017).

Child development experts emphasized that to optimize academic outcomes, the environment needs to target both cool and hot executive functions by nurturing the social, emotional, physical, and cognitive abilities of children (Diamond and Ling, 2016; Bierman et al., 2017). In 2010, Diamond argued toward a whole-child approach by addressing skills and attitudes instead of content. In particular, she stated that the most efficient and cost-effective ways to enhance children’s academic outcomes are to focus on academic, social, emotion, and physical development. To this vein, physical health can also be enhanced by targeting emotional, social, and cognitive wellness (Diamond, 2010). Nearly a decade later, Tomporowski and Pesce (2019) advocated that skills acquisition is the common underlying mechanism during training of motor and cognitive tasks, evident in exercise, sports, and performance arts. When dual tasks (i.e., motor and cognitive) are performed simultaneously, mental processes activated may enhance declarative memory (Tomporowski and Qazi, 2020).

Physical activity participation and motor skill development offer substantial benefits for preschool children’s physical, motor, cognitive, and psychosocial development (Bart et al., 2007; Piek et al., 2008; Carson et al., 2017). Benefits of physical activity in preschool children include improved motor development and fitness, as well as bone and skeletal health (Carson et al., 2017). Physical activity in children and adolescents has been shown to improve academic achievement, student engagement, executive function skills, and metacognition (Owen et al., 2016; Álvarez-Bueno et al., 2017a,b). Recent recommendations by the World Health Organization (2019) suggest that preschool children should spend at least 180min in a variety of types of physical activity at any intensity, of which at least 60min is moderate-to-vigorous intensity physical activity, spread throughout the day. Children with high levels of actual and perceived physical competence (i.e., ability or perceived ability to perform motor tasks) are more likely to engage in higher level of physical activity, with mutual benefits on both areas of physical competence and activity (Stodden et al., 2008; Barnett et al., 2015).

Early childhood centers are important settings because children spend a large amount of their waking hours there (OECD, 2017). However, physical activity is becoming compromised as children are mostly engaged in sedentary activities (O’Brien et al., 2018). The integration of physical activity in the academic classroom has received increased interest by educators, researchers, and professional organizations (Institute of Medicine, 2013; ASCD, 2014; Webster et al., 2015), and the number of intervention programs has rapidly increased during the last decade (Vazou et al., 2020). A body of research on movement integration (i.e., physically active lessons or active breaks and cognitively engaging physical activity) has shown physical and cognitive benefits in children and adolescents (e.g., academic achievement and on-task behavior; Watson et al., 2017; Daly-Smith et al., 2018; Bedard et al., 2019; Norris et al., 2020).

Research in the area of cognitively engaging physical activity is leaning toward the qualitative characteristics (e.g., task novelty, complexity, and selection of mental strategies for problem-solving), rather than the quantitative aspects (i.e., dose, intensity, and duration) of physical activity (Pesce, 2012). Current empirical evidence is mainly targeting cool executive functions. For instance, in a six-week program, 14 kindergarten classes were randomly assigned to one of the three experimental conditions (Schmidt et al., 2020): In the physical-cognitive condition, children were engaged with games combining physical and cognitive demands. These games were adapted from original common games (e.g., Simon says) but inherently included training of cool executive function skills (e.g., rule changes and response to target stimuli and inhibit from non-target stimuli). The cognitive condition included the same game with the physical-cognitive condition, but children were engaged with fine motor movements of light intensity. Finally, children in the control condition did not alter their usual daily practice. Results showed that the physical-cognitive and cognitive conditions elicited improvements on children’s updating performance, with children reporting equal levels of enjoyment, but no changes on inhibition and shifting. In the “Red Light, Purple Light” program, preschool children practiced inhibition with physically active games during circle time. Greater gains were found in self-regulation and academic achievement over the preschool year for the intervention group (Schmitt et al., 2015), as well as significant gains in letter-word identification after an 8-week implementation period (Tominey and McClelland, 2011).

Few physical activity programs have included hot executive functions: For instance, the “Animal Fun” program with duration of 6months imitated animal movements to enhance preschool children’s social and behavioral outcomes (Piek et al., 2015). Children’s prosocial behavior and inattention were improved after 6months and maintained after 18months of follow-up. Acute positive effects on verbal and social engagement in the classroom were evident after structured physical activity lessons that challenged executive functions and social-emotional skills, compared to the non-physically active days in preschoolers (Vazou et al., 2017). Other programs, including sport games, found less peer-relationship and emotional problems as well as higher scores in prosocial behaviors of preschool children (Griffith et al., 2010). Physical play in the classroom was found to be positively related to emotional competence (i.e., peer relationships) in boys (Linsey and Colwell, 2003).

Overall, despite the strong consensus among educators, researchers, and policy makers that education should have a more holistic approach with equal focus on cognitive, social-emotional, and physical development (ASCD, 2014; Bierman et al., 2017), classroom-based physical activity programs targeting explicitly psychosocial, cognitive and physical development, are lacking. Physical activity programs, including both cool and hot executive functions in early education, are scarce (Diamond and Lee, 2011; Vazou et al., 2019). This is one of the very few studies designed in the preschool classroom, combining a whole-child approach, integrating physical activity with academic content, executive functions, and social-emotional skills (self-regulation, self- and social awareness, and relationships skills). The program focused on providing enjoyable and cognitively engaging physical activities targeting motor skill development, as well as regulation of executive function and social–emotional skills. Concomitantly, social environment is fundamental in influencing feelings of competence and social acceptance, which is also, impacting motivation and behavior (Harter, 1978).

The present feasibility study was designed to help researchers and practitioners determine whether the “Move for Thought (M4T) preK-K” program should be recommended for a larger scale and potentially implemented as a physical activity program on preschool children’s motor, socio-emotional, and cognitive skills. Our study goals were aligned with current literature emphasizing the importance of early examinations of the feasibility of interventions with a focus on acceptability, demand, implementation, practicality, and limited-efficacy testing (Bowen et al., 2009). Specifically, the first purpose was to examine the feasibility and usability of the M4T preK-K program on children’s physical and cognitive engagement during the preschool day. The second purpose was to assess effectiveness of the program by examining changes in children’s attention and behavioral control, social skills, executive functions, and perceived motor skill competence, from the beginning to the end of the intervention period. It was expected that children would manifest improved social and cognitive skills as well as perceived motor skill competence and executive functions, at the end of the implementation of the M4T preK-K program, compared to the control group.

## Materials and Methods

### Participants

**Figure 1:**
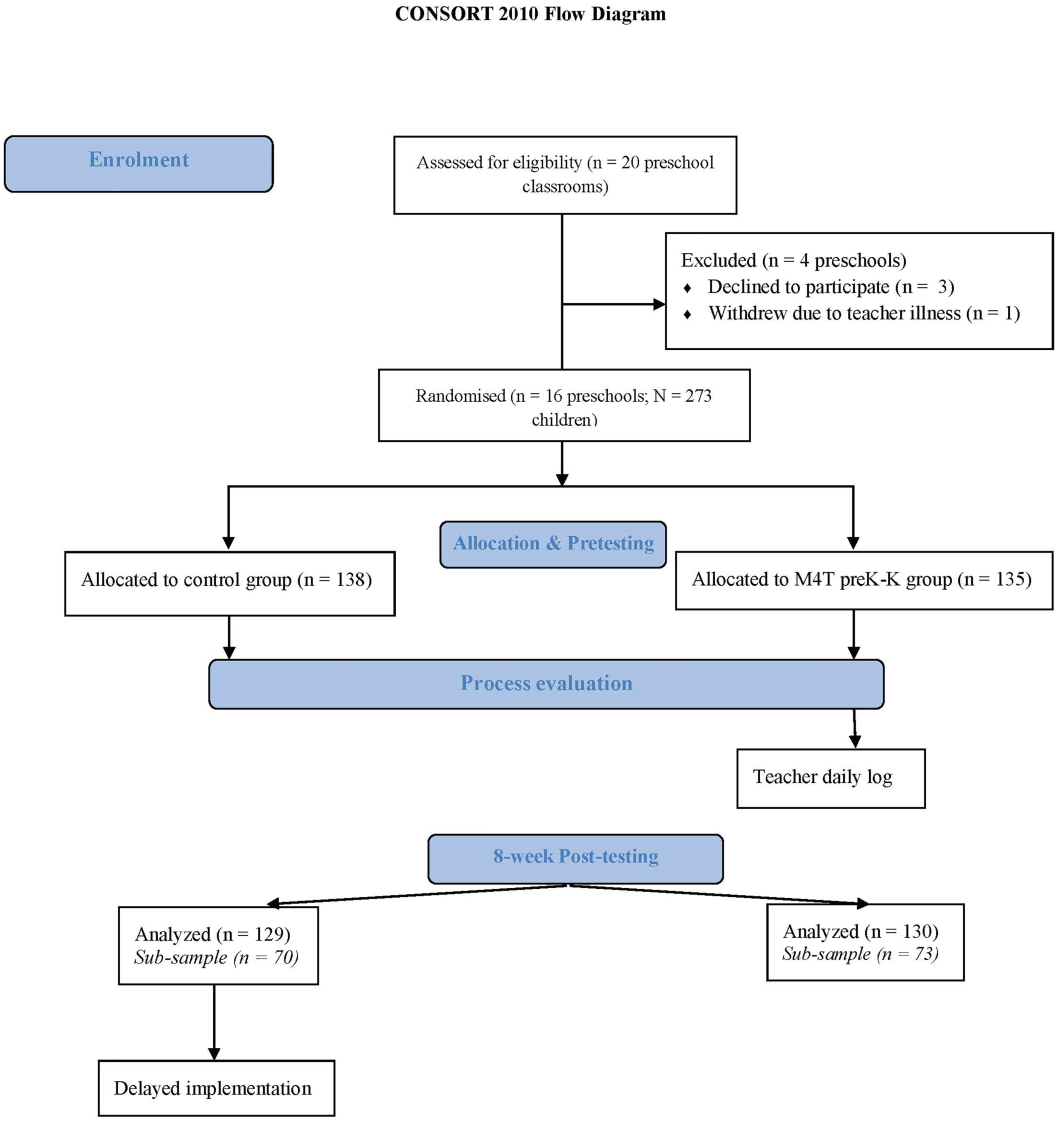
Flow Chart of Participants.

Participants included 273 preschool children 3–5years and their early childhood educators (n=16) from 16 preschool classrooms across the State of Iowa. Preschool education is not mandatory in the United States. Children were randomly assigned to the intervention (8 classrooms, 138 children) or control conditions (8 classrooms, 135 children). Some outcomes (EF and perceived motor competence) were collected with a subsample from the existing sample, consisting of 141 children from 4 control (*n*=73, *M*_age_=4.00 *SD*=0.61) and 4 intervention preschool classrooms (*n*=70, *M*_age_=4.34 *SD*=0.62). The study was exempt from the State University Institutional Review Board as the activities were offered as part of the regular instructional strategies by teachers and no children were identified in the data collection (student list was coded by the teachers). Teacher’s and school’s consent were obtained before the implementation of the program. Children’s assent was obtained before each testing. Parents were informed about the study and could have their child opt out from the data collection if desired. Children’s and teachers’ demographic characteristics are presented in Table 1 whereas the flow of participants in portrayed in Figure 1.

**Table 1:** Baseline characteristics of study sample.

Characteristics	Intervention (*n*=130)	Control (*n*=129)	*t*-test	*p*
Child age (in years), mean (SD)	4.31 (0.61)	4.13 (0.60)		
Child female participants, n (%)	63 (48.5%)	61 (47.2%)		
Preschools	4	6		
Classrooms (No of teachers)	8	8		
Female teachers	7	8		
Age of teacher (years), mean (SD)	36.5 (7.67)	41 (15.07)	0.57	0.464
Race of teacher	100% White	100% White		
Years teaching preschool, mean (SD)	10.25 (9.03)	9.94 (7.20)	0.006	0.940
Size of preschool	3 small schools (4–7 staff)	3 small schools (3 staff)		
N children in classroom	16.50	17.75		
N children per preschool, mean (SD)	84.12 (58.09)	85.37 (58.88)	0.002	0.967
Low parental income^*^	49%	75%	1.34	0.271
Full-day program	6 classes	7 classes		
Headstart classes^*^	2	3		
PA throughout preschool day	120.63min (37.74)	131.25min (73.77)	0.132	0.722

PA, physical activity

* as an indicator of low socioeconomic status.

### Experimental Design

This study involved a randomized controlled trial at the school level. Randomization occurred after the pretest assessments using a computer-based algorithm by an independent researcher. Children were not aware of the purpose of the study or the experimental conditions. Implementation was done at the class level by the preschool teacher during regular classroom activities and school hours. All teachers were blind to the conditions and were informed that they would receive access to the M4T preK-K resources either immediately or at a later time due to limited research support (i.e., the control group received delayed implementation upon completion of the program for the intervention group).

### Procedure

Outcome measures were assessed by early childhood educators at pretest and at the end of 8 weeks for both groups. The full sample was assessed by early childhood educators on attention, behavioral control, assertion, cooperation, and self-control. Moreover, a subsample of children was assessed on inhibition and perceived motor skill competence at the preschool center individually by trained research assistants blinded to the experimental conditions.

Upon completion of the pretest measures, the intervention group received a printed copy of the M4T preK-K resources (book with the activities, CD with the M4T preK-K music, supporting academic materials, such as flashcards with pictures from the recommended literacy books) and a printed copy of the daily teacher log. An 1 h of training (webinar) was provided by the research team to the intervention group to become familiar with the program and the questionnaires. During the implementation period, early childhood educators in the intervention group completed the teacher log daily whenever they included a M4T preK-K activity during the preschool day. In the control group, teachers were instructed to continue with their usual practices without any changes in their regular instructional format.

#### Intervention

The teachers in the intervention group were instructed to use one cognitively engaging physical activity per day from the M4T preK-K program, for eight consecutive weeks during fall and before the holiday break. No further instructions were provided as the goal was for teachers to have the autonomy to select which, when, and how to integrate the physical activities during the school day, based on their students’ needs and their own level of comfort. This approach was followed to increase external and ecological validity by evaluating the real-world feasibility of the intervention. The M4T preK-K program was developed for children in the preschool and kindergarten environment and includes a total of 57 activities for large group, small group, and transitions in the classroom, as well as outdoor activities for large play areas, without the need of expensive equipment apart from the already existing ones in the preschool and kindergarten environment (e.g., popular age-appropriate children’s books, scarves, beanbags, small balls, tape, and hula hoops). The duration of the activities can vary based on the level of integration and the goals of the teacher, with some activities being very short (2–3min; e.g., transitions), some lasting for 15–20min (e.g., when explaining concepts like what comes first, next, and last in a story or a book), while the majority of the activities aimed to last about 10min. All activities have recommendations for progression and for additional challenges. The M4T preK-K program is freely available on the website of Iowa State University.^1^

**Figure 2:**
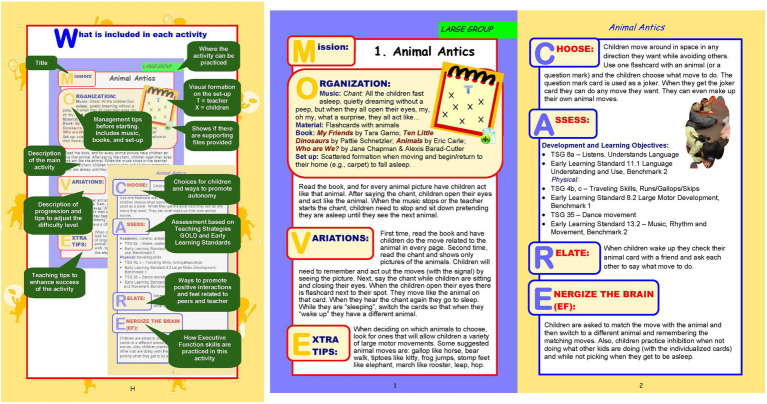
Example of “Move for Thought” preK-K Activities.

Each activity in the M4T preK-K program was designed to assist in meeting physical activity needs, improving physical literacy and fundamental gross motor skills (locomotor, non-locomotor, and manipulative skills) and adopt a “whole-child” approach by practicing children’s physical, cognitive, social, and emotional skills. More specifically, in the M4T preK-K program, each activity aimed to help children: (1) achieve fundamental motor skill goals through modification and developmentally appropriate progression after practice and learning, (2) practice and be assessed in physical, cognitive, socio-emotional and academic domain, (3) explore rhythmic and musical concepts through singing and dancing in original music, (4) explore age-appropriate popular books and develop positive experiences with math, literacy, and learning, (5) feel autonomous by encouraging them to have choices and self-exploration, (6) self-regulate by identifying emotions, practicing self-control, cognitive flexibility, and responsible decision making, and (7) develop social awareness and relationship skills by cooperating, sharing, and communicating with others through positive interactions with peers and their teachers. Figure 2 (panels A and B) provides an example of the structure and components of the M4T preK-K activities for each skill area. As shown in the figure, each activity includes the MOVE and CARE acronyms that refer to: MOVE-Mission (name of activity), Organization (instructions for activity; e.g., set up and equipment required), Variations (suggestions for progression in more complex motor tasks), Extra tips to keep children motivated. CARE stands for: Choose (different options to support autonomy), Assess (assessments based on preschool learning standards), Relate (recommendations for training peer social awareness and relationship skills among children), and Energize the brain (examples to train executive functions).

### Measures

Demographic information was received *via* a questionnaire completed by teachers. The full sample of children was assessed on:

#### Attention and Behavioral Control in the Classroom

Attention (9 items) and Behavioral Control (i.e., 9 items) in the classroom was measured using the 18-item Strength and Weaknesses of ADHD symptoms and normal behavior (SWAN) questionnaire (Swanson et al., 2012). Teachers responded on a 7-point Likert scale ranging from 1 “far below average” to 7 “far above average” for each student. Example questions include “settle down and rest (control constant activity)” for behavioral control and “Gives close attention to detail and avoids careless mistakes” for attention. The SWAN questionnaire has been distributed in a number of population-based studies with clinical and typical development groups, producing normal distributions in school-based studies, and having high internal consistency (*α*=0.94–0.95; Swanson et al., 2012). It has also been shown to be appropriate from children as young as preschool (Lakes et al., 2012). Cronbach’s α coefficients of internal consistency were high (*α*=0.94–0.95) for both factors.

#### Hot Executive Function Skills

Cooperation, Assertion, and Self-Control in the classroom was used using the 30-item Social Skills Rating Scale questionnaire (Gresham and Elliot, 1990). The Cooperation subscale includes behaviors such as helping others, sharing materials, and complying with rules. The Assertion subscale includes initiating behaviors, such as asking others for information, and responding to the actions of others. The Self-control subscale includes behaviors that emerge in conflict situations (e.g., responding to teasing) and in non-conflict situations (e.g., taking turns and compromising). Teachers responded on a 3-point Likert scale ranging from 1 “never” to 3 “very often.” Example questions include “participates in game” and “follows directions” (i.e., cooperation), “invites others” and “make friends” (i.e., assertion), and “waits turns” and “accepts peer ideas” (i.e., self-control). This questionnaire has been previously used in preschool children (Frey et al., 2011). In the current study, the alpha coefficient of internal consistency was high for all factors (*α*=0.87–0.94).

The subsample was also assessed on:

#### Cool Executive Function Skills

The computerized Stroop-like “DayNight” (Gerstadt et al., 1994) was used to measure inhibition and task switching. The test consisted of a set of pictures showing either a picture of the moon or the picture of the sun. Children were instructed to say “day” to a picture of moon and “night” to a picture of sun (e.g., “When you see the picture of a sun, I want you to say “night”). During this task, the child must inhibit and switch simultaneously to provide the correct answer without receiving any feedback. The task included 2 practice trials (up to 3 practice sessions if the child misses either trial in practice) and 16 testing trials (half are sun pictures and half are moon pictures). The stimulus presentation time was 1500ms. Children’s answers were recorded with a microphone and were processed using the Audacity software. Children received 1 point for correct responses, with an aggregated score for accuracy.

#### Perceived Movement Skill Competence

The “Pictorial scale for Perceived Movement Skill Competence (PMSC) for young children” (Barnett et al., 2016) tool was used. The 12-item questionnaire assesses six locomotor (run, gallop, hop, leap, horizontal jump, and slide) and six object control skills (striking a stationary ball, stationary dribble, catch, overhand throw, and underhand roll). A pictorial plate presentation format is used, comprising of two gender-specific pictures displayed side by side. The pictures depict one child competent in a particular task and a child who is not. Children are asked to choose the picture that represents them the most (e.g., picture that looks more like him/her). If children choose the competent picture, they are asked “are you really good at ….?.” Children’s responses score in a 4-point Likert scale between 1, “poorly skilled child” to 4, “highly skilled child.” Cronbach’s alpha coefficient of internal consistency was acceptable (*α*=0.81, 0.77, for pre and post-test, respectively).

### Process Evaluation

#### Daily Teacher Log

Session fidelity in the intervention group was recorded by early childhood educators on a daily basis. If there were no M4T preK-K activities on a particular day, the daily log was not completed. The daily long included questions regarding: (1) name, frequency, and duration of M4T preK-K activities, (2) area the activity was conducted, and (3) teacher overall experience from the M4T preK-K program, which was broken down into:

1. Teacher and child satisfaction on the M4T preK-K activities, on 5-point scale (1, “very unsatisfied” to 5, “very satisfied”).
2. Children’s physical and cognitive engagement during M4T preK-K activities, on a 3-point scale (1, “not at all” to 3, “very”).
3. Evidence of children’s engagement regarding working with others, regulating emotions, and exhibiting self-control (“yes” or “no” checklist).

Upon completion of the implementation period, teacher overall reflection in implementing the M4T program was measured. Using a 5-point scale (1=not at all to 5=very much), teacher’s ability to: (1) lead physical activities in the classroom, (2) teach motor skills, (3) implement the M4T preK-K program, and (4) implement variations of the activities (progression) were reported by teachers. Further, teachers were asked whether they believed they needed additional training on how to use the M4T preK-K activities during the implementation period, using the same response scale.

### Statistical Analyses

Data derived from the teacher logs were analyzed with Excel and are presented descriptively. Statistical analyses were conducted using IBM SPSS (version 26), and alpha level was set at *p*<0.05 for the quantitative data. Mixed ANOVAs were conducted with two groups (intervention vs. control) for the between-subject factor and two time points (pre vs. post) for the within-subject factor, separately for each outcome. To account for the nesting nature of the data, follow-up analyses of the significant outcomes were conducted using linear mixed models in IBM SPSS Statistics, version 26.0 (2010 SPSS Inc., IBM Company). Linear mixed models adjusted for clustering at the class level were used to assess the impact of the group (M4T, AB or control), time (treated as categorical with levels baseline and 8weeks), and the group-by-time interaction. A random intercept was used to account for the repeated measures of each participant. Cohen’s d provided a measure of effect size (adjusted difference between intervention and control group over time divided by the pooled standard deviation of change; Vacha-Haase and Thompson, 2004). Cohen’s *d*=0.2 were considered small, *d*=0.5 medium, *d*=0.8 as large effect sizes (Cohen, 1988; Vacha-Haase and Thompson, 2004).

## Results

A summary of the detailed descriptive statistics of the outcomes is presented on Table 2.

**Table 2:** Means (and standard deviations) per outcome as a function of condition.

	Move for Thought				Control			
Variables	N	Pre	Post	Post-pre ES	N	Pre	Post	Post-pre ES
		M (SD)	M (SD)			M (SD)	M (SD)	
Attention^a^ *(SWAN)*	129	4.12 (0.74)	4.36 (0.76)	0.32	128	4.21 (0.81)	4.21 (0.76)	0.00
Behavioral Control^a^ *(SWAN)*	129	4.07 (0.75)	4.24 (0.81)	0.22	128	4.26 (0.90)	4.18 (0.75)	−0.10
Hot Executive Functions Assertion^b^ *(SSRS)*	130	2.35 (0.68)	2.43 (0.62)	0.12	129	2.40 (0.66)	2.42 (0.76)	0.03
Cooperation^b^ *(SSRS)*	130	2.69 (0.56)	2.70 (0.53)	0.02	129	2.73 (0.47)	2.70 (0.47)	−0.06
Self-Control^b^ *(SSRS)*	130	2.49 (0.61)	2.51 (0.63)	0.03	129	2.67 (0.49)	2.66 (0.49)	−0.02
Cool Executive Function^c^ *(DayNight)*	53	10.94 (4.59)	11.93 (4.16)	0.23	52	10.39 (4.77)	12.00 (4.00)	0.37
Perceived FMS^d^ *(PMSC)*	55	3.35 (0.56)	3.36 (0.50)	0.02	47	3.40 (0.47)	3.53 (0.50)	0.27

M, mean; SD, Standard deviation; Post-pre Effect Size (ES), Cohen’s d; FMS, Fundamental movement skills.

a 5-point Likert scale.

b 3-point Likert scale.

c accuracy scores.

d 4-point Likert scale.

### Mixed Analyses of Variance

#### Attention

Both the main effect of time [*F*(1, 255)=13.00, *p*<0.001, *η*^2^=0.048] and interaction between time and condition [*F*(1, 255)=11.18, *p*<0.001, *η*^2^=0.028] on children’s attention scores were found significant. The main effect of condition [*F*(1, 255)<1, *p*=0.734] was not significant. At post-test, both groups improved their scores; however, the intervention group outperformed the control group. After accounting for the nesting nature of the data, the interaction between time and condition on children’s attention was significant for the intervention group (Adjusted mean difference=0.26, 95% CI 0.06 to 0.45, *p*=0.013) but not for the control group (Adjusted mean difference=0.03 CI −0.17 to 0.23, *p*=0.757).

#### Behavioral Control

The main effects of time [*F*(1, 255)=1.36, *p*=0.245] and condition [*F*(1, 255)<1, *p*=0.496] on children’s behavioral control scores were not significant. However, the interaction between time and condition [*F*(1, 255)=11.71, *p*<0.001, *η*^2^=0.044] was significant. At post-test, the intervention group performed better than the control. After accounting for the nesting nature of the data, the interaction between time and condition on children’s behavioral control was not significant (Adjusted mean difference=0.24, 95% CI −0.04 to 0.52, *p*=0.094).

#### Hot Executive Function Skills

There were no main effects of time [*F*(1, 257)<1, *p*=0.439] and condition [*F*(1, 257) <1, *p*=0.743] on children’s cooperation skills. In addition, the interaction between and time and condition was not significant [*F*(1, 257)=1.04, *p*=0.310].

There were no main effects of time [*F*(1, 257)=2.4, *p*=0.122] and condition [*F*(1, 257) <1, *p*=0.778] on children’s assertion skills. Also, the interaction between time and condition was not significant [*F*(1, 257)<1, *p*=0.364].

The main effects of time [*F*(1, 257)<1, *p*=0.859] and interaction between time and condition [*F*(1, 257)<1, *p*=0.447] were not significant on self-control skills. However, the main effect of condition [*F*(1, 257)=6.48, *p*=0.012] was statistically significant. Regardless of time, the intervention group had lower scores on self-control, compared to the control group.

#### Cool Executive Function Skills

The main effect of time was found to be significant [*F*(1, 103)=8.78, *p*=0.004]. However, the main effect of condition [*F*(1, 103)<1, *p*=0.744] and the interaction between time and condition were not significant [*F*(1, 103)<1, *p*=0.471].

#### Perceived Fundamental Movement Skills

The main effects of time [*F*(1, 100)=1.44, *p*=0.233], condition [*F*(1, 100)=1.80, *p*=0.182], and interaction between time and condition [*F*(1, 100)=1.25, *p*=0.267] were not significant.

### Process Evaluation

#### Teacher Log Data

All early childhood educators (8/8) returned the daily teacher log. They reported that the dose of the intervention was implemented as intended on average 98% (*SD*=0.10), meaning once per day. The average duration of each session was 10.65min (*SD*=3.17). Early childhood educators reported high satisfaction with the program and activities (*M*=4.23/5, *SD*=0.45), as well as high satisfaction from the children (*M*=4.12/5, *SD*=0.69). Early childhood educators reported that children were both physically (*M*=2.81/3, *SD*=0.12) and cognitively engaged (*M*=2.59/3, *SD*=0.35) during the M4T preK-K activities. Finally, teachers reported that children during the M4T sessions were given several opportunities to exhibiting self-control (*M*=85%, *SD*=0.14), whereas the focus on regulating emotions (*M*=35%, *SD*=0.67) and working with others (*M*=42%, *SD*=0.29) was less evident.

**Figure 3:**
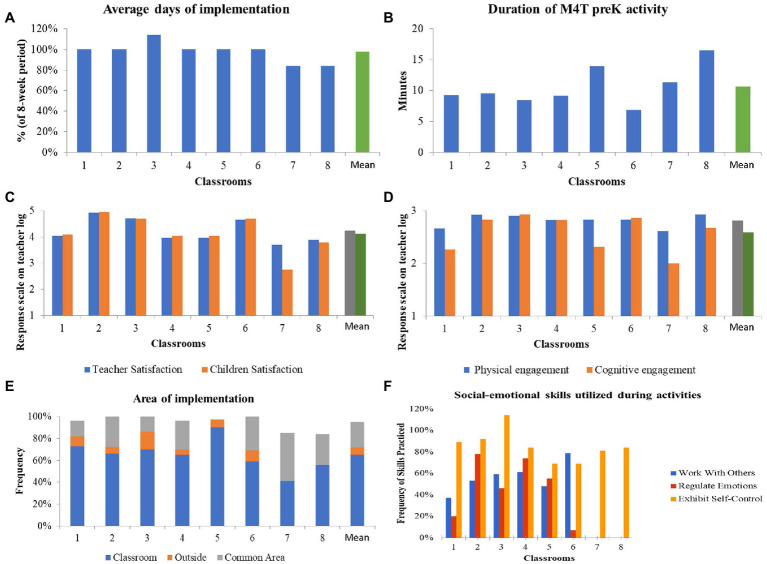
Process Evaluation Results for Intervention Class. Panel A: Days of Implementation, Panel B: Average Duration of a M4T preK-K Activity, Panel C: Teacher and Student Satisfaction from Implementation, Panel D: Level of Physical and Cognitive Engagement during Implementation, Panel E: Area of Implementation, and Panel F: Social-Emotional Skills (Working with Others, Regulating Emotions, Exhibiting Self-Control) Practiced During Implementation.

The majority of the activities were conducted inside the classroom (*M*=65%, *SD*=0.14). Alternatively, activities were conducted in the common area (*M*=23%, *SD*=0.13), while only few occurred outside (*M*=7%, *SD*=0.05). One-fourth of the times the early childhood educators used the supporting files (*M*=22%, *SD*=0.17), and about one-third of the lessons incorporated the M4T preK-K music (*M*=31%, *SD*=0.15). Detailed responses per class are portrayed in Figure 3. Further, teachers’ confidence in their ability to teach motor skills and PA in the classroom, in general, was 4.5/5 (*SD*=0.53), to implement the M4T preK-K program was 4.43 (*SD*=0.53), and to implement variation of the M4T preK-K (as a progression) was 4.37 (*SD*=0.52). Lastly, the teachers reported low to somewhat need for additional training on the M4T preK-K program (*M*=2.12, *SD*=0.64).

## Discussion

This study examined the feasibility, usability, and effectiveness of an 8-week intervention study in preschool children with the M4T preK-K program that integrated physical activity with academic content, executive function, social, and emotional skills. The study targeted improving children’s attention and behavioral control, social skills, executive functions, and perceived motor skill competence, which are acknowledged as crucial for school readiness and academic success. Results showed that children’s attention was significantly improved after the intervention, compared to the control group. However, behavioral control, social skills, inhibition on a computerized task, and perceived motor competence were not improved for both groups. In addition, intervention fidelity results showed that the implementation of short (i.e., around 10min) M4T preK-K sessions daily was a feasible and realistic goal for preschool children, who were both physically and cognitively engaged.

Confirming our hypotheses, children’s attention performance improved significantly after participating in cognitively engaging physical activities, compared to the control group. However, children’s behavioral control scores remained unaffected. The high levels of cognitive engagement were also evident in the teachers’ daily log, reporting that during the M4T preK-K sessions, children were both physically and cognitively engaged. These results are in line with previous literature, supporting that integrating physical activity during learning process can contribute to cognitive benefits in children without compromising academic time (Mavilidi et al., 2018, 2019). For example, a series of studies found improved learning outcomes in preschool children when integrating physical activity during several academic domains, such as language, geography, math, and science (Mavilidi et al., 2015, 2016, 2017, 2018; Toumpaniari et al., 2015). Notably, the preschool environment is typically the first structured environment for young children, so training programs that help children exhibit higher levels of self-regulation in the classroom are beneficial for school readiness and constitute an aspirational goal in the educational system (Greenberg and Weissberg, 2018).

The M4T preK-K program was predicated on the idea that providing physical activities in the classroom in integration with cognitive (literacy books and academic content) and motor skills is both appropriate and beneficial for the children’s development. Emerging evidence suggests that we should shift the focus of quantitative characteristics (e.g., dose, intensity, and frequency) toward qualitative characteristics of physical activity (e.g., movement coordination, type, novelty, and complexity of movement, activation of mental strategies; Pesce, 2012; Vazou et al., 2019). In particular, the role of motor competence in the exercise-cognition interplay is essential (van der Fels et al., 2015; Tomporowski and Pesce, 2019).

In this study, children’s perceptions of motor skill competence were measured for object control and locomotor skills, as practiced in the M4T preK-K program, with no significant changes for both groups. The absence of a significant change in perhaps unsurprising considering the self-reported nature of the scale and that the sample was a little younger than the typical age the scale has been used before (*M*_age_=4.2, *SD*=0.60, in this study, whereas *M*_age_=4.7, *SD*=0.46 and *M*_age_=6.5, *SD*=0.8, in the Barnett et al., 2015, 2016, respectively). Children that young tend to have relatively undifferentiated and exaggerated perceptions of competence as they lack extensive experience in applying those skills in games, which might result in low validity of the scale. The lack of a clear perception on motor skill competence may have led to a ceiling effect, as evidenced by the very high scores of 3.35–3.55 on a 4-point scale (with *SD*=0.50). Overcoming this limitation of the study, future research can consider (1) the use of objectively measures of motor skill competence and (2) the use of accelerometers to account for the intensity of physical activities (Cliff et al., 2009).

Importantly, the current study was consisted of cognitively engaging physical activity in the form of challenging games with continuous change of rules and instructions with simultaneous focus on preacademic concepts. Research shows that these qualitative characteristics may transform physical activity into meaningful learning experiences for cognitive development (Pesce et al., 2019). Cognitively engaging physical activity which combines motor and cognitive tasks may act as brain stimulators, explicitly training skills, such as executive functions (Diamond and Ling, 2016; Pesce and Ben-Soussan, 2016). For example, a recent study in preschool children found improvements in updating performance after participating in games consisting cognitively engaging physical activity (Schmidt et al., 2020). However, even though there were significant changes on attention, contrary to our hypothesis, there were no significant differences between groups on inhibitory control, as measured with the subsample. All children regardless of their group improved on the sun/moon task from the beginning to the end of the 8-week period, which likely reflects a learning effect, possibly modulated by the environment and experience. The non-significant differences among groups could be attributed to the small subsample size, which is a limitation of this study, possible methodological challenges with the task due to the young age of the sample (e.g., accurate measure of perceived competence in young children), and/or insufficient dose of implementation. A replication of this finding with a large sample and with a longer intervention (more weeks) is warranted.

Of note, the vast body of current research focuses mostly on executive functions, with rare examples existing targeting hot executive functions (Diamond and Lee, 2011; Vazou et al., 2017; Pesce et al., 2020). For example, a mindful martial arts training program occurring two-three 45-min sessions/week for 3months in children from kindergarten through Grade 5 found improvements on their cognitive, affective, and physical self-regulation, as well as classroom conduct and prosocial behavior (Lakes and Hoyt, 2004). The present study included training of children’s social and emotional self-regulation skills. However, contrary to previous literature, we did not find any changes between the control and intervention group at end of the program (Lakes and Hoyt, 2004; Diamond et al., 2007; Piek et al., 2015; Vazou et al., 2017). Assertion and cooperation skills remained stable for both groups whereas self-control skills, as well as inhibitory control skills, improved for both groups.

As reported by early childhood educators, fidelity check evaluations confirmed that teachers targeted more on children’s physical and cognitive engagement and less on practicing their social and emotional skills (see Figure 3, panel F). In fact, in some classes, activities engaging children with “regulating emotions” and “working with others” were absent. “Exhibiting self-control” was practiced more often. If early childhood educators did not engage children’s social and emotional skills as intended per protocol, it is logical to assume that there could not be improvements after the end of the program on such skills. Interestingly, this program provided with instructions and variations to task complexity based on children’s responses and progress level as increased scaffold. However, early childhood educators chose to implement the easiest options of the activities. Possibly, early childhood educators lack deeper understanding on improving social and emotional skills in integration with physical activity in preschool children or lack time to explore and progress on the more advanced versions of the activities without any guidance or support. The training included in this study was very limited as we wanted to explore the level of feasibility of the program based on teachers’ existing experience and knowledge. Hence, it is evident that future research should incorporate more explicit guidance through concrete examples (e.g., video-based resources) to support teachers on progression and implementation of these components.

Another possible explanation for the null findings of this study on hot and cool executive function skills could be attributed to the short duration of the program. Previous studies had substantially longer durations (e.g., 3months, Lakes and Hoyt, 2004; 6months with sustained effects at 18-month follow-up, Piek et al., 2015; 1–2years; Diamond et al., 2007). Of note, the post-intervention effect sizes of the intervention group are small but positive compared to the pre-intervention values, whereas in the control group are smaller for assertion (M4T preK-K ES=0.12 vs. control ES=0.02), and negative for cooperation and self-control. Longer duration of the program may have been able to provoke larger effects.

Finally, another purpose of the study was to examine the feasibility and usability of the M4T preK-K program. The results based on the daily teacher logs were positive. The duration of M4T preK-K program was well-received by early childhood educators with the dose of the physical activity sessions delivered as intended (98% and an average duration of approximately 10min daily). Both early childhood educators and children reported high levels of satisfaction with the program (Figure 3). These findings are very encouraging rendering this approach as an effective solution for overcoming teacher barriers related to lack of time (Naylor et al., 2015). Furthermore, this program was found to be feasible, with potential for future acceptability, adaptation, and implementation by early childhood educators.

Despite the fact that this study focused on the feasibility and effectiveness of the M4T preK-K program on preschoolers, the activities expand to the Kindergarten years. Programs with cognitively engaging physical activity aiming at kindergarten children may offer a smoother transition to the school environment. Previous research found kindergarten to be more responsive to improvements on attention and behavioral control compared to preschool and 2nd grade children (Vazou et al., 2021). Further examination of the feasibility and effectiveness of the program with kindergarteners is warranted. Additionally, a replication of the study with a larger sample in a cluster randomized controlled trial of longer duration should be considered in the future. It is important to note that the small sample size of this study (especially for the subsample) limits statistical power and restricts generalizability to the broader population of preschoolers. Notably, the significant results on children’s attention scores were completed by the not-blinded teachers, while measures which were executed by blinded research assistants were not significant. Another limitation of the study was the measurement of the outcome variables with the teacher reported scales, presenting challenges for possible biases in providing desirable responses, even though such scales are acceptable in research involving preschool children. Overall, intervention programs in children need to be tailored based on children’s age, skill levels, and cognitive development.

Nevertheless, an undisputable strength of the present study is its inclusion of both cool and hot executive function skills. To the best of our knowledge, there is no other such physical program existing for preschool children in the academic classroom. In particular, the emphasis of the present study on enhancing preschool children’s cognitive, motor, social, and emotional skills through integrating physical activity during the classroom instruction provides a unique contribution to the field. Programs usually include only one element, most commonly cool executive functions, whereas programs on hot executive functions are scarce.

Diamond (2015, p.963) proposed that interventions that improve executive functions: (1) train and challenge diverse motor and executive function skills, (2) bring joy, pride, and self-confidence, and (3) provide a sense of belonging. Concomitantly, training of executive function skills has been shown to be more enjoyable and effective when is incorporated in everyday activities and games (Takacs and Kassai, 2019). The current program included all these components and is aligned with self-determination theory, advocating that relatedness, competence, and autonomy are comprising the basic psychological needs, contributing to intrinsic motivation and positive motivational outcomes (e.g., commitment, effort; Ryan and Deci, 2000). Indeed, there was a clear focus of the program on team work and collaboration.

Lastly, the inclusion of evaluation of the implementation and delivery of the program constitute another strength of the present study. The findings from fidelity checklists showed that pragmatic implementation of the program was successful, permitting for greater confidence in generalizing the findings to real-world school settings. A recent systematic review showed that around half of the interventions with movement integration were researcher-led, undermining their possibilities for sustainability, dissemination, and scalability (Vazou et al., 2020). It was concluded that regarding delivery of interventions, researchers should be more consistent on reporting fidelity.

## Conclusion

Physical activity programs could serve as the basis of a holistic approach to child development, supporting not only physical health but also cognitive, motor, social, and emotional benefits in children (Diamond and Ling, 2016; Carson et al., 2017). The present study was well-received by early childhood educators and children, providing evidence of the feasibility and usability of a physical activity program integrating with academic instruction, with potential benefits on children’s cognitive, social, and emotional skills. Promoting interventions in early childhood is fundamental for school readiness, academic performance, and success in career and life in the long-term (Moffitt et al., 2011).

## Data Availability Statement

The raw data supporting the conclusions of this article will be made available by the authors, without undue reservation.

## Ethics Statement

The studies involving human participants were reviewed and approved by Iowa State University. Written informed consent from the participants legal guardian/next of kin was not required to participate in this study in accordance with the national legislation and the institutional requirements.

## Author Contributions

SV: contributed in all steps of the study (conceptualization, methodology, supervision, analysis, and writing). MM: analysis, writing, and interpretation of the results. All authors reviewed and approved the final manuscript.

## Conflict of Interest

The authors declare that the research was conducted in the absence of any commercial or financial relationships that could be construed as a potential conflict of interest.

## Publisher’s Note

All claims expressed in this article are solely those of the authors and do not necessarily represent those of their affiliated organizations, or those of the publisher, the editors and the reviewers. Any product that may be evaluated in this article, or claim that may be made by its manufacturer, is not guaranteed or endorsed by the publisher.

## Footnotes

^1^ www.moveforthought.org

## References

[ref1] Álvarez-Bueno C. Pesce C. Cavero-Redondo I. Sánchez-López M. Garrido-Miguel M. Martínez-Vizcaíno V. (2017a). Academic achievement and physical activity: a meta-analysis. Pediatrics 140:e20171498. doi: 10.1542/peds.2017-1498, PMID: 29175972

[ref2] Álvarez-Bueno C. Pesce C. Cavero-Redondo I. Sanchez-Lopez M. Martínez-Hortelano J. A. Martinez-Vizcaino V. (2017b). The effect of physical activity interventions on children’s cognition and metacognition: A systematic review and meta-analysis. J. Am. Acad. Child Adolesc. Psychiatry 56, 729–738. doi: 10.1016/j.jaac.2017.06.012, PMID: 28838577

[ref3] ASCD (2014). Whole School, Whole Child, Whole Community: A Collaborative Approach to Learning and Health. United States: Alexandria.

[ref4] Barnett L. M. Morgan P. J. Van Beurden E. Ball K. Lubans D. R. (2011). A reverse pathway? Actual and perceived skill proficiency and physical activity. Med. Sci. Sports Exercise 43, 898–904. doi: 10.1249/MSS.0b013e3181fdfadd, PMID: 20962694

[ref5] Barnett L. M. Ridgers N. D. Zask A. Salmon J. (2015). Face validity and reliability of a pictorial instrument for assessing fundamental movement skill perceived competence in young children. J. Sci. Med. Sport. 18, 98–102. 24485803 10.1016/j.jsams.2013.12.004

[ref6] Bart O. Hajami D. Bar-Haim Y. (2007). Predicting school adjustment from motor abilities in kindergarten. Infant Child Dev. Int. J. Res. Practice 16, 597–615. doi: 10.1002/icd.514

[ref7] Bedard C. St John L. Bremer E. Graham J. D. Cairney J. (2019). A systematic review and meta-analysis on the effects of physically active classrooms on educational and enjoyment outcomes in school age children. PLoS One 14:e0218633. doi: 10.1371/journal.pone.0218633, PMID: 31237913 PMC6592532

[ref8] Bierman K. L. Greenberg M. T. Abenavoli R. (2017). Promoting Social and Emotional Learning in Preschool: Programs and Practices that Work. State College, PA: Pennsylvania State University, Edna Bennet Pierce Prevention Research Center.

[ref9] Blair C. Ursache A. (2011). “A bidirectional model of executive functions and self-regulation” in Handbook of Self-Regulation: Research, Theory, and Applications. eds. Vohs K. D. Baumeister R. F. (New York: Guilford Press), 300–320.

[ref10] Bodrova E. Leong D. J. (2006). Tools of the Mind. Australia: Pearson Australia Pty Limited.

[ref11] Bowen D. J. Kreuter M. Spring B. Cofta-Woerpel L. Linnan L. Weiner D.. (2009). How we design feasibility studies. Am. J. Prev. Med. 36, 452–457. doi: 10.1016/j.amepre.2009.02.002, PMID: 19362699 PMC2859314

[ref12] Bull R. Espy K. A. Wiebe S. A. (2008). Short-term memory, working memory, and executive functioning in preschoolers: longitudinal predictors of mathematical achievement at age 7 years. Dev. Neuropsychol. 33, 205–228. doi: 10.1080/87565640801982312, PMID: 18473197 PMC2729141

[ref13] Bull R. Scerif G. (2001). Executive functioning as a predictor of children's mathematics ability: inhibition, switching, and working memory. Dev. Neuropsychol. 19, 273–293. doi: 10.1207/S15326942DN1903_3, PMID: 11758669

[ref14] Carson V. Lee E. Y. Hewitt L. Jennings C. Hunter S. Kuzik N.. (2017). Systematic review of the relationships between physical activity and health indicators in the early years (0-4 years). BMC Public Health 17, 33–63. doi: 10.1186/s12889-017-4860-0 29287590 PMC5747177

[ref15] Cliff D. P. Reilly J. J. Okely A. D. (2009). Methodological considerations in using accelerometers to assess habitual physical activity in children aged 0–5 years. J. Sci. Med. Sport 12, 557–567. doi: 10.1016/j.jsams.2008.10.008, PMID: 19147404

[ref16] Cohen J. (1988). Statistical Power Analysis for the Behavioral Sciences. 2nd *Edn*. Hillsdale: Erlbaum.

[ref17] Daly-Smith A. J. Zwolinsky S. McKenna J. Tomporowski P. D. Defeyter M. A. Manley A. (2018). Systematic review of acute physically active learning and classroom movement breaks on children’s physical activity, cognition, academic performance and classroom behaviour: understanding critical design features. BMJ open Sport Exercise Med. 4, 1–16. doi: 10.1136/bmjsem-2018-000341 PMC5884342 29629186

[ref600] Denham S. A. Brown C. (2010). “Play nice with others”: Social-emotional learning and academic success. Early. Educ. Dev. 21, 652-680.

[ref18] Diamond A. (2010). The evidence base for improving school outcomes by addressing the whole child and by addressing skills and attitudes, not just content. Early Educ. Dev. 21, 780–793. doi: 10.1080/10409289.2010.514522, PMID: 21274420 PMC3026344

[ref19] Diamond A. (2013). Executive functions. Annu. Rev. Psychol. 64, 135–168. doi: 10.1146/annurev-psych-113011-143750, PMID: 23020641 PMC4084861

[ref20] Diamond A. Barnett W. S. Thomas J. Munro S. (2007). Preschool program improves cognitive control. Science 318, 1387–1388. doi: 10.1126/science.1151148, PMID: 18048670 PMC2174918

[ref21] Diamond A. (2015). Effects of physical exercise on executive functions: going beyond simply moving to moving with thought. Annals of Sports Med. Res. 2:1011. PMID: 26000340 PMC4437637

[ref22] Diamond A. Lee K. (2011). Interventions shown to aid executive function development in children 4 to 12 years old. Science 333, 959–964. doi: 10.1126/science.1204529, PMID: 21852486 PMC3159917

[ref23] Diamond A. Ling D. S. (2016). Conclusions about interventions, programs, and approaches for improving executive functions that appear justified and those that, despite much hype, do not. Dev. Cogn. Neurosci. 18, 34–48. doi: 10.1016/j.dcn.2015.11.005, PMID: 26749076 PMC5108631

[ref24] Durlak J. A. Weissberg R. P. Dymnicki A. B. Taylor R. D. Schellinger K. B. (2011). The impact of enhancing students’ social and emotional learning: A meta-analysis of school-based universal interventions. Child Dev. 82, 405–432. doi: 10.1111/j.1467-8624.2010.01564.x, PMID: 21291449

[ref500] Eisenberg N. Valiente C. Eggum N. D. (2010). Self-regulation and school readiness. Early Educ. Dev. 21, 681–698. doi: 10.1080/10409289.2010.497451 PMC3018834 21234283

[ref25] Frey J. R. Elliott S. N. Gresham F. M. (2011). Preschoolers’ social skills: advances in assessment for intervention using social behavior ratings. Sch. Ment. Heal. 3, 179–190. doi: 10.1007/s12310-011-9060-y

[ref26] Garon N. (2016). A review of hot executive functions in preschoolers. J. Self-Reg. 2, 57–80. doi: 10.11588/josar.2016.2.34354

[ref27] Gerstadt C. L. Hong Y. J. Diamond A. (1994). The relationship between cognition and action: performance of children 3 1/2-7 years old on a Stroop-like day-night test. Cognition 53, 129–153. doi: 10.1016/0010-0277(94)90068-X, PMID: 7805351

[ref28] Greenberg M. Weissberg R. (2018). Social and Emotional Development Matters: Taking Action Now for Future Generations. United States: Edna Bennett Pierce Prevention Research Center, Pennsylvania State University.

[ref29] Gresham F. M. Elliot S. N. (1990). Manual for the Social Skills Rating System. Circle Pines, MN: American Guidance Service.

[ref30] Griffith L. J. Dowda M. Dezateux C. Pate R. (2010). Associations between sport and screen-entertainment with mental health problems in 5-year-old children. Int. J. Behav. Nutr. Phys. Act. 7, 30–11. doi: 10.1186/1479-5868-7-30 20409310 PMC2867988

[ref31] Happaney K. Zelazo P. D. Stuss D. T. (2004). Development of orbitofrontal function: current themes and future directions. Brain Cogn. 55, 1–10. doi: 10.1016/j.bandc.2004.01.001, PMID: 15134839

[ref32] Harter S. (1978). Effectance motivation reconsidered. Toward Dev. Model. Hum. Dev. 21, 34–64.

[ref33] Institute of Medicine (2013). Educating the Student Body: Taking Physical Activity and Physical Education to School. Washington, DC: The National Academies Press. 24851299

[ref34] Lakes K. D. Hoyt W. T. (2004). Promoting self-regulation through school-based martial arts training. J. Appl. Dev. Psychol. 25, 283–302. doi: 10.1016/j.appdev.2004.04.002

[ref35] Lakes K. D. Swanson J. M. Riggs M. (2012). The reliability and validity of the English and Spanish strengths and weaknesses of ADHD and normal behavior (SWAN) rating scales: continuum measures of hyperactivity and inattention. J. Atten. Disord. 16, 510–516. doi: 10.1177/1087054711413550, PMID: 21807955 PMC3575190

[ref36] Leshem R. De Fano A. Ben-Soussan T. D. (2020). The implications of motor and cognitive inhibition for hot and cool executive functions: The case of quadrato motor training. Front. Psychol. 11:940. doi: 10.3389/fpsyg.2020.00940 32508720 PMC7250031

[ref37] Linsey E. W. Colwell M. J. (2003). “Preschoolers' emotional competence: links to pretend and physical play.” Child Study Journal. Gale OneFile: Health and Medicine, Available at: link.gale.com/apps/doc/A110262692/HRCA?u=anon~8d83071d&sid=googleScholar&xid=119bfe25(Accessed October 26, 2021).

[ref38] Mavilidi M. F. Okely A. D. Chandler P. Cliff D. P. Paas F. (2015). Effects of integrated physical exercises and gestures on preschool children’s foreign language vocabulary learning. Educ. Psychol. Rev. 27, 413–426. doi: 10.1007/s10648-015-9337-z

[ref39] Mavilidi M. F. Okely A. D. Chandler P. Paas F. (2016). Infusing physical activities into the classroom: effects on preschool children's geography learning. Mind Brain Educ. 10, 256–263. doi: 10.1111/mbe.12131

[ref40] Mavilidi M. F. Okely A. D. Chandler P. Paas F. (2017). Effects of integrating physical activities into a science lesson on preschool children's learning and enjoyment. Appl. Cogn. Psychol. 31, 281–290. doi: 10.1002/acp.3325

[ref41] Mavilidi M. F. Okely A. Chandler P. Domazet S. L. Paas F. (2018). Immediate and delayed effects of integrating physical activity into preschool children’s learning of numeracy skills. J. Exp. Child Psychol. 166, 502–519. doi: 10.1016/j.jecp.2017.09.009, PMID: 29096234

[ref42] Mavilidi M. Ouwehand K. Okely A. D. Chandler P. Paas F. (2019). “Embodying learning through physical activity and gestures in preschool children,” in Advances in Cognitive Load Theory: Rethinking Teaching. eds. Tindall-Ford S. Agostinho S. Sweller J. (New York: Routledge), 103–118.

[ref43] Moffitt T. E. Arseneault L. Belsky D. Dickson N. Hancox R. J. Harrington H.. (2011). A gradient of childhood self-control predicts health, wealth, and public safety. Proc. Natl. Acad. Sci. U. S. A. 108, 2693–2698. doi: 10.1073/pnas.1010076108, PMID: 21262822 PMC3041102

[ref700] Nadeem E. Maslak K. Chacko A. Hoagwood K. E. (2010). Aligning Research and Policy on Social-Emotional and Academic Competence for Young Children. Early. Educ. Dev. 21, 765–779. doi: 10.1080/10409289.2010.497452 25632216 PMC4306577

[ref44] Naylor P. J. Nettlefold L. Race D. Hoy C. Ashe M. C. Higgins J. W.. (2015). Implementation of school based physical activity interventions: a systematic review. Prev. Med. 72, 95–115. doi: 10.1016/j.ypmed.2014.12.034, PMID: 25575800

[ref45] Norris E. van Steen T. Direito A. Stamatakis E. (2020). Physically active lessons in schools and their impact on physical activity, educational, health and cognition outcomes: a systematic review and meta-analysis. Br. J. Sports Med. 54, 826–838. doi: 10.1136/bjsports-2018-100502, PMID: 31619381

[ref46] O’Brien K. T. Vanderloo L. M. Bruijns B. A. Truelove S. Tucker P. (2018). Physical activity and sedentary time among preschoolers in Centre-based childcare: a systematic review. Int. J. Behav. Nutr. Phys. Act. 15:117. doi: 10.1186/s12966-018-0745-6, PMID: 30463585 PMC6249856

[ref47] OECD (2017). Building skills for all in Australia. Policy insights from the survey of adult skills. Available at: https://www.oecd-ilibrary.org/sites/9789264281110-en/index.html?itemId=/content/publication/9789264281110-en (Accessed September 28, 2021).

[ref48] Owen K. B. Parker P. D. Van Zanden B. MacMillan F. Astell-Burt T. Lonsdale C. (2016). Physical activity and school engagement in youth: a systematic review and meta-analysis. Educ. Psychol. 51, 129–145. doi: 10.1080/00461520.2016.1151793

[ref49] Pesce C. (2012). Shifting the focus from quantitative to qualitative exercise characteristics in exercise and cognition research. J. Sport Exerc. Psychol. 34, 766–786. doi: 10.1123/jsep.34.6.766, PMID: 23204358

[ref50] Pesce C. Ben-Soussan T. D. (2016). ““Cogito ergo sum” or “ambulo ergo sum”? New perspectives in developmental exercise and cognition research” in Exercise-cognition interaction: Neuroscience perspectives. ed. McMorris T. (London: Elsevier Academic Press), 251–282.

[ref51] Pesce C. Croce R. Ben-Soussan T. D. Vazou S. McCullick B. Tomporowski P. D.. (2019). Variability of practice as an interface between motor and cognitive development. Int. J. Sport Exercise Psychol. 17, 133–152. doi: 10.1080/1612197X.2016.1223421

[ref52] Pesce C. Lakes K. D. Stodden D. F. Marchetti R. (2020). Fostering self-control development with a designed intervention in physical education: A two-year class-randomized trial. Child Dev. 92, 937–958. doi: 10.1111/cdev.13445 32840871

[ref53] Piek J. P. Bradbury G. S. Elsley S. C. Tate L. (2008). Motor coordination and social–emotional behaviour in preschool-aged children. Int. J. Disabil. Dev. Educ. 55, 143–151. doi: 10.1080/10349120802033592

[ref54] Piek J. P. Kane R. Rigoli D. McLaren S. Roberts C. M. Rooney R.. (2015). Does the animal fun program improve social-emotional and behavioural outcomes in children aged 4–6 years? Hum. Mov. Sci. 43, 155–163. doi: 10.1016/j.humov.2015.08.004, PMID: 26298689

[ref55] Ryan R. M. Deci E. L. (2000). The darker and brighter sides of human existence: basic psychological needs as a unifying concept. Psychol. Inq. 11, 319–338. doi: 10.1207/S15327965PLI1104_03

[ref56] Schmidt M. Mavilidi M. F. Singh A. Englert C. (2020). Combining physical and cognitive training to improve kindergarten children’s executive functions: A cluster randomized controlled trial. Contemp. Educ. Psychol. 63:101908. doi: 10.1016/j.cedpsych.2020.101908

[ref57] Schmitt S. A. McClelland M. M. Tominey S. L. Acock A. C. (2015). Strengthening school readiness for head start children: evaluation of a self-regulation intervention. Early Child. Res. Q. 30, 20–31. doi: 10.1016/j.ecresq.2014.08.001

[ref58] Stodden D. F. Goodway J. D. Langendorfer S. J. Roberton M. A. Rudisill M. E. Garcia C.. (2008). A developmental perspective on the role of motor skill competence in physical activity: An emergent relationship. Quest 60, 290–306. doi: 10.1080/00336297.2008.10483582

[ref59] Swanson J. M. Schuck S. Porter M. M. Carlson C. Hartman C. A. Sergeant J. A.. (2012). Categorical and dimensional definitions and evaluations of symptoms of ADHD: history of the SNAP and the SWAN rating scales. Int. J. Edu. Psychol. Assessment 10, 51–70. PMID: 26504617 PMC4618695

[ref60] Takacs Z. K. Kassai R. (2019). The efficacy of different interventions to foster children’s executive function skills: A series of meta-analyses. Psychol. Bull. 145, 653–697. doi: 10.1037/bul0000195, PMID: 31033315

[ref61] Tominey S. L. McClelland M. M. (2011). Red light, purple light: findings from a randomized trial using circle time games to improve behavioral self-regulation in preschool. Early Educ. Dev. 22, 489–519. doi: 10.1080/10409289.2011.574258

[ref62] Tomporowski P. D. Pesce C. (2019). Exercise, sports, and performance arts benefit cognition via a common process. Psychol. Bull. 145, 929–951. doi: 10.1037/bul0000200, PMID: 31192623

[ref63] Tomporowski P. D. Qazi A. S. (2020). Cognitive-motor dual task interference effects on declarative memory: A theory-based review. Front. Psychol. 11:1015. doi: 10.3389/fpsyg.2020.01015 32670130 PMC7326112

[ref64] Toumpaniari K. Loyens S. Mavilidi M. F. Paas F. (2015). Preschool children’s foreign language vocabulary learning by embodying words through physical activity and gesturing. Educ. Psychol. Rev. 27, 445–456. doi: 10.1007/s10648-015-9316-4

[ref65] Vacha-Haase T. Thompson B. (2004). How to estimate and interpret various effect sizes. J. Couns. Psychol. 51, 473–481. doi: 10.1037/0022-0167.51.4.473

[ref66] van der Fels I. M. te Wierike S. C. Hartman E. Elferink-Gemser M. T. Smith J. Visscher C. (2015). The relationship between motor skills and cognitive skills in 4–16-year-old typically developing children: A systematic review. J. Sci. Med. Sport 18, 697–703. doi: 10.1016/j.jsams.2014.09.007, PMID: 25311901

[ref67] Vazou S. Long K. Lakes K. D. Whalen N. L. (2021). “Walkabouts Integrated Physical Activities from Preschool to Second Grade: Feasibility and Effect on Classroom Engagement” in Child and Youth Care Forum. Vol. 50 (US: Springer), 39–55.

[ref68] Vazou S. Mantis C. Luze G. Krogh J. S. (2017). Self-perceptions and social–emotional classroom engagement following structured physical activity among preschoolers: A feasibility study. J. Sport Health Sci. 6, 241–247. doi: 10.1016/j.jshs.2016.01.006, PMID: 30356608 PMC6189013

[ref69] Vazou S. Pesce C. Lakes K. Smiley-Oyen A. (2019). More than one road leads to Rome: a narrative review and meta-analysis of physical activity intervention effects on cognition in youth. Int. J. Sport Exercise Psychol. 17, 153–178. doi: 10.1080/1612197X.2016.1223423, PMID: 31289454 PMC6615761

[ref70] Vazou S. Webster C. A. Stewart G. Candal P. Egan C. A. Pennell A.. (2020). A systematic review and qualitative synthesis resulting in a typology of elementary classroom movement integration interventions. Sports Medi 6, 1–16. doi: 10.1186/s40798-019-0218-8, PMID: 31907711 PMC6944721

[ref71] Watson A. Timperio A. Brown H. Best K. Hesketh K. D. (2017). Effect of classroom-based physical activity interventions on academic and physical activity outcomes: a systematic review and meta-analysis. Int. J. Behav. Nutr. Phys. Act. 14, 1–24. doi: 10.1186/s12966-017-0569-9 28841890 PMC5574081

[ref72] Webster C. A. Russ L. Vazou S. Goh T. L. Erwin H. (2015). Integrating movement in academic classrooms: understanding, applying and advancing the knowledge base. Obes. Rev. 16, 691–701. doi: 10.1111/obr.12285, PMID: 25904462

[ref73] World Health Organization (2019). Guidelines on physical activity, sedentary behaviour and sleep for children under 5 years of age. Retrieved from Available at: https://www.who.int/news/item/24-04-2019-to-grow-up-healthy-children-need-to-sit-less-and-play-more 31091057

